# Development, improvement and funding of the emergency medicine cases open-access podcast

**DOI:** 10.5116/ijme.57f8.c1b4

**Published:** 2016-10-22

**Authors:** Lucas B. Chartier, Anton Helman

**Affiliations:** 1 Emergency Department, University Health Network, Toronto, ON, Canada; 2 Emergency Department, North York General Hospital, Toronto, ON, Canada

**Keywords:** Development, improvement and funding, emergency medicine, open-access podcast

## Introduction

Internet-based medical education resources, such as podcasts, have increased in number and popularity in recent years, and are being integrated into all levels of medical training and continuing medical education.^1^^,^^2^ Podcasts may be more effective than traditional methods of instruction such as textbooks for learners who prefer auditory modes of learning.^3^ Social media, through which podcasts can be distributed, is defined as the social interaction among people through virtual communities. The term ‘Free Open-Access Medical EDucation' (FOAMed), coined in 2012, is a subset of social media relating to the “creation and exchange of user-generated [medical] content via virtual networks and communities”.^4^ It is growing rapidly, out of a desire by health care professionals for free medical education and as a means to stay current with the wide scope of EM literature.^5^^,^^6^ This article describes the creation of the Emergency Medicine Cases (EMC) podcast, as well as the innovative transformation that occurred as a result of its conversion to a free open-access resource and its partnership with an academic institution.

### Description of the Podcast

EMC, which was created in 2010, is targeted at health care professionals working in the EM environment. In a monthly podcast, the host (ADH) poses clinical questions to guest experts, discussing current controversies and describing evidence-based treatments. Episodes cover broad-based and clinically relevant topics from atrial fibrillation to cognitive decision-making. Eighty-two three-hour podcasts have been produced to date, each accompanied by a written summary, and 46 five-minute ‘Best Case Ever’ capsules.

Initially, EMC was offered on a subscription-only basis. As of March 2014, 1,680 health care providers had subscribed to EMC. As demand for free online education increased, however, a free open-access model was adopted in April 2014. To cover the significant ongoing costs of the program, EMC partnered in July 2014 with a non-profit academic institution in Toronto, Canada, dedicated to advancing the care of patients requiring emergency services. The social media presence of EMC was also increased.

### Impact of the Partnership

Since adopting a free model, EMC’s website traffic has increased almost six-fold, with the average number of monthly visitors increasing from approximately 3,000 prior to over 17,000 after the transition. The average number of monthly downloads of podcasts increased from 11,000 to approximately 90,000 over the same period, and is continuously growing. There was also an increase in international listeners, with listeners from Canada, the United States and Australia composing the majority of all listeners.

Feedback from listeners suggests EMC is a valued resource, and preliminary data show promising results. In the first four months following the transition to free access, 248 listeners voluntarily filled out a survey on the EMC website. Based on answering ‘agree’ or ‘strongly agree’ on a 5-point Likert scale, 89% of respondents felt that what they learned from an episode would change their practice, 91% felt they would be more confident the next time they saw a patient with the discussed condition, and 99% would recommend the episode to a colleague.

EMC’s true impact is difficult to evaluate, but not more so than traditional publications. Citation rate and journal impact factors imperfectly reflect the true impact of a given article or journal. Access to academic journals is often limited by firewalls, and the end-users – clinicians treating patients – may not immediately benefit from the information. This contributes to the very lengthy time from article publication to widespread integration into clinical practice.^4^ By contrast, hundreds of thousands of downloads of EMC episodes suggest widespread interest and knowledge translation in real time.

To our knowledge, EMC is the only free-access EM podcast wholly funded and supported by a non-profit academic institution. The transition to free access has contributed to the increase in EMC’s accessibility, universality and immediacy. The support provided by the academic partnership has enabled EMC to exist as a free resource, as well as to increase its accountability and quality. The significant increase in website traffic, podcast downloads, and international users attest to the increased reach afforded by free access education. Most important, however, is the immediacy of the knowledge translation. The processes required for scholarly article and textbook publications are lengthy, and content is often said to represent how medicine was practiced two and five years prior to their publication, respectively.^4^ By contrast, the process of planning, recording and publishing podcasts takes days or weeks. Free access has also allowed EMC to advertise and promote episodes more rapidly than ever before, with immediate uptake by end-users.

Bandiera and colleagues have argued that academic medical centers should fund educational programs in order to protect the time of the educators, and thereby increase the reputation of both.^7^ Our academic collaboration demonstrates exactly that. While the peer review process of journal articles relies on the expertise of a select few, EMC is supported by dozens of academic educators and accountable to thousands of listeners who can freely comment or criticize the product in real time. The partnership helped develop a community of practice, which can crowd-source topics of interest, recommend experts, and curate content. It has also made possible many related academic endeavours: a series of interactive simulation-based videos with peer-reviewed expert commentary, a series of free eBooks based on EMC’s content, and a Global Emergency Medicine Journal Club, a novel initiative featuring interviews with the lead authors of landmark EM studies. The latter is presented in a podcast format, along with crowd-sourced critiques and opinions of the studies.

## Conclusions

Social media constitute an ever-increasing aspect of continuing medical education. There are many advantages for podcasts and educational programs to pursue a free open-access model, such as accessibility, universality and immediacy. In developing novel free education resources, medical educators should consider financial partnership as part of their long-term planning for sustainability. Operating costs pose a formidable barrier to entry for other would-be podcast producers. Most of EMC’s recent success, as well as improvement in quality and accountability, would not have been possible without the financial and consultative support of an academic institution. In the future, the currency for academic advancement may shift from traditional journal publication towards global impact through new venues such as podcasts. In order to ensure that the benefits of free access online medical education are sustained over time however, we must ensure that we fund and support these important innovations. 

### Acknowledgements

The authors thank their colleagues Drs. Bjug Borgundvaag, Shirley Lee, Rick Penciner, and Ms. Sasha Chapman for their critical review and thoughtful comments on earlier versions of this manuscript.

### Conflict of Interest

The authors are the creators, authors and editors of the Emergency Medicine Cases podcast being discussed in the manuscript. ADH receives direct financial support for the podcast from the Schwartz/Reisman Emergency Medicine Institute, of which he is the Education Innovation Lead.
